# Operational strategies for achieving comprehensive cancer center community outreach and engagement objectives: impact and logic models

**DOI:** 10.1186/s13690-024-01252-1

**Published:** 2024-03-14

**Authors:** Folakemi T. Odedina, Manisha Salinas, Monica Albertie, Doug Murrell, Kristin Munoz

**Affiliations:** 1 Community Outreach and Engagement Office and Programs, Mayo Clinic Comprehensive Cancer Center, 4500 San Pablo Road, Jacksonville, FL 3224 USA; 2 Community Outreach and Engagement Office and Programs, Mayo Clinic Comprehensive Cancer Center, Phoenix, AZ USA; 3 Community Outreach and Engagement Office and Programs, Mayo Clinic Comprehensive Cancer Center, Rochester, MN USA

**Keywords:** Health disparities, Cancer, Community outreach, Community engagement, Evidence-based practice

## Abstract

**Background:**

The National Cancer Institute’s (NCI) Cancer Center Support Grants (CCSGs) encourages Cancer Centers to address health disparities and reduce the cancer burden in their Catchment Area (CA) through an organized Community Outreach and Engagement (COE) structure. This paper shares the development of two guide models that fosters the operations of the Mayo Clinic Comprehensive Cancer Center (MCCCC) COE Office and programs, the MCCCC COE Impact Model and the MCCCC COE Logic Model.

**Methods:**

Following a less than stellar CCSG rating for COE in 2018, the MCCCC developed a transition team to specifically address the critique and create a transformative plan for engaging communities to address cancer burden in the CA. A qualitative research approach was employed, focusing on organizing and displaying the relationship between MCCCC COE processes and outcomes through impact and logic models. An impact model was developed to illustrate the components of the CCSG and connect those components to short- and long-term COE outcomes. A logic model was developed to track and monitor activities for continuous process improvement for all COE activities.

**Results:**

The impact and logic model serve as a roadmap to monitor progress towards short- and long-term COE goals of the MCCCC. The COE operational strategies draw upon bidirectional partnership, evidence-based practices, and research facilitation to respond to the CCSG critique.

**Conclusion:**

These strategies demonstrate successful practices in addressing cancer burden, promoting health equity and eliminating cancer disparities in the MCCCC CA.

**Table:** 

Text box 1. Contributions to the literature
• Addressing population health to achieve health quality for all requires community outreach and engagement, which is very difficult to operationalize. A guide framework or model, developed in partnership with the community, can effectively assist cancer centers and other healthcare institutions to be responsive to the needs of the communities they serve.
• Working closely with members of the community that we serve, we developed two models that have guided our activities in minoritized and marginalized communities. The Impact Model provides a roadmap towards achieving short-term and long-term goals. The Logic Model provides a framework to evaluate the community outreach and engagement activities, improve program effectiveness, and inform decisions about future program development.
• The Impact and Logic Models developed in this report can be easily adapted by other cancer centers and institutions working in minoritized and marginalized communities to address health disparities.

## Background

Community outreach and engagement (COE) is one of the notable components of the United States (U.S) National Cancer Institute (NCI) Cancer Center Support Grants (CCSGs). Given that COE is the gold standard in addressing health disparities and closing the health gaps in minoritized and marginalized communities, COE is regarded as the “center” of NCI-designated Cancer Centers and the cornerstone of Cancer Centers. As a CCSG Component [1], the “*primary metric in evaluating the strength of COE is the scope, quality, and impact of the center’s community outreach and engagement activities on the burden of cancer in the Center’s stated catchment area”.* Based on this metric, the NCI encourages all NCI-designated Cancer Centers to foster and share “COE knowledge-base, best practices, and tools” for adoption, adaptation and implementation by other NCI-designated cancer centers, the scientific community and COE communities for the advancement of the progress against the burden of cancer and cancer risk factors. Meeting or exceeding the CCSG requirements for COE is not an easy task and involves long-term commitment to a bi-directional and equal partnership between a Cancer Center and communities in its designated Catchment Area (CA). As defined by NCI, CA is a “*self-defined geographic area that the Center serves or intends to serve in the research it conducts, the communities it engages, and the outreach it performs*” [1]. COE’s success starts with a clear definition of the CA(s) as defined by the cancer center, followed by knowledge of the cancer-related characteristics of the CA communities, and full engagement of the communities to address their cancer needs and facilitate research that is responsive to the cancer needs of the community, including inclusive clinical trials.

Evidence of cancer center COE’s responsiveness to the needs of the CA communities can be documented through seven (7) criteria raised in the CCSG application guidelines [1]:

1. Clear definition and justification of the CA communities.
2. Understanding of the characteristics and determinants of the cancer burden in the CA communities.
3. Impactful COE infrastructure, resources and activities focused on cancer prevention, control and research in the CA communities.
4. Center’s engagement of center members on community needs to catalyze responsive high-impact research.
5. Assisting with accrual to clinical trials from the center’s CA.
6. Effective cancer control efforts that impact cancer burden in the catchment area communities.
7. Expansion of reach beyond CA for national and global impact.

Unfortunately, there is limited guidance on COE operational strategies to help cancer centers meet these criteria. The primary objective of this paper is to share two guide models that were developed to foster the operations of the Mayo Clinic Comprehensive Cancer Center (MCCCC) COE Office and Programs, the MCCCC COE Impact Model and the MCCCC COE Logic Model.

## Methods

In 2018, the MCCCC COE CCSG component received a less than stellar rating with the primary weakness that “*it was unclear how these* (COE) *projects were informed by the community and community advisory boards and how they are responsive to the needs of minorities in the catchment areas*”. Additionally, the reviewers noted that the disparity in rural cancer outreach, screening, control, and survivorship was not fully addressed. This review was a wake-up call for the MCCCC, with a clear mandate that COE would need to rise above its challenges to effectively address the CCSG critiques. With new leadership at both the cancer center level and the COE level, a COE transition team was formulated to address the 2018 CCSG critiques.

The primary objective of this general qualitative inquiry was to develop guide models that would support the implementation of effective COE activities within the CAs of the MCCCC. Since our goal was to change our activities and processes for better outcomes, our primary focus was to develop a theory of change with a framework that would organize and show the relationship between our processes and outcomes. To create the roadmap for the change, our qualitative research design focused on impact and logic models design [2] to qualitatively demonstrate the causal relationships between the COE planned work and intended outcomes [3].

The study settings were the CAs of the MCCCC, including Arizona, Florida and the Midwest. The objective was met through the engagement of the Community Advisory Board (CAB) and a COE transition team. The primary mission of the CAB is to serve as a liaison between the MCCCC and the CA communities by advising the COE and MCCCC leadership and enhancing the relationship between the MCCCC and the community. In this way, they build both the academic and community capacity to facilitate community-based research in partnership, sharing knowledge and disseminating information to improve health outcomes. Each site CAB (AZ, FL and Midwest) has 8–10 members and is comprised of survivors, lay caregivers, local cancer advocates and national/regional representatives. For this project, we held listening sessions with each CAB to obtain feedback on the programs and activities of the COE. The feedback was integrated into the activities of the COE transition team.

The COE transition team comprised MCCCC scientists and clinicians across the three sites. The COE transition team was charged with developing a plan to address the critiques and develop a transformative strategic plan through 5-year and 2-year objectives, building on the CAB feedback. Led by the MCCCC COE Enterprise Deputy Director, supported by the COE faculty advisors and COE Operations Team, and in collaboration with the CAB, three working groups were formed and met regularly to develop a strategic plan that would address the 2018 CCSG critiques for COE. The working group members met over six months in 2022. Additionally, CAB meetings were organized for gathering direct community feedback consistently. After the completion of these sessions, working group reports were presented to the MCCCC leadership team for input and final approval. The transition team’s endeavors led to the creation of innovative strategies for COE, including 2-year and 5-year goals. Drawing from these strategies, the COE team subsequently crafted both an impact model and a logic model based on the newly developed strategic plan.

## Results

### The MCCCC COE Impact Model

**Fig. 1:**
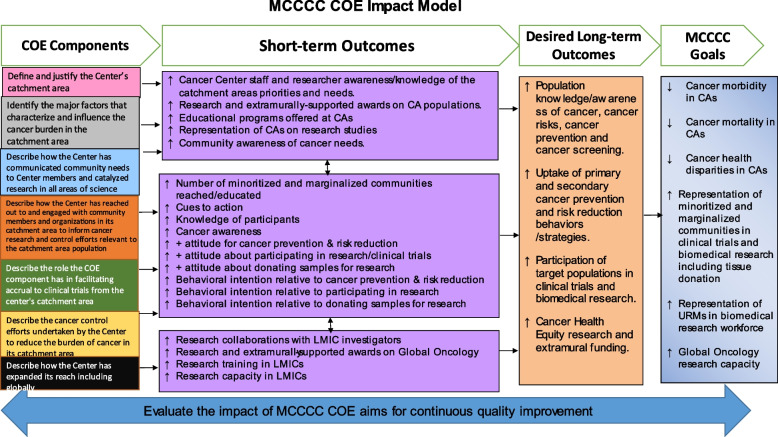
Mayo Clinic Comprehensive Cancer Center Community Outreach & Engagement (MCCCC COE) Impact Model to describe specific processes and outcomes to meet overall MCCCC goals

In developing the COE Impact Model (COE-IM), we adopted a business approach of a functional model that clearly connects the CCSG COE criteria to the MCCCC goals through COE short-term and long-term outcomes (see Fig. 1). The COE-IM is anchored by the CCSG COE requirements [1] and the MCCCC goals for its CA communities. The CCSG requirements [1] for COE include:

- Define and justify the center’s CA.
- Identify the major factors that characterize and influence the cancer burden in the CA.
- Describe how the center has reached out to and engaged with community members and organizations in its CA to inform cancer research and control efforts of relevance to the CAs.
- Describe how the center has communicated community needs to center members and catalyzed research in all areas of science.
- Describe the role the COE component has in facilitating accrual to clinical trials from the center’s CA.
- Describe the cancer control efforts undertaken by the center to reduce the burden of cancer in its CA.
- Describe how the center has expanded its reach, including globally.

With a vision for a cancer center of the future, the mission of the MCCCC is a center without walls building upon all Mayo Clinic strengths, delivering cancer expertise to the patients and communities served by MCCCC anywhere, anytime and for anyone. MCCCC strives towards this mission by connecting patients and communities with data, intercepting pre-cancer, developing more effective treatments, transforming cancer care delivery and transforming cancer clinical trials to achieve six population health goals: (1) decreasing cancer morbidity in MCCCC CAs, (2) decreasing cancer mortality in MCCCC CAs, (3) decreasing cancer health disparities in MCCCC CAs, (4) increasing the representation of minoritized and marginalized communities in clinical trials and biomedical research, including tissue donation, (5) increasing the representation of underrepresented minorities (URMs) in biomedical research workforce, and (6) increasing global oncology research capacity. In alignment with the MCCCC goals, the long-term objectives of the COE are to increase:

- Population knowledge/awareness of cancer, cancer risks, cancer prevention and cancer screening.
- The uptake of primary and secondary cancer prevention and risk reduction behaviors /strategies.
- Participation of target populations in clinical trials and biomedical research.
- Cancer-related health equity research and extramural funding.

To complete the COE-IM, short-term objectives that will facilitate the achievement of COE long-term objectives were developed in alignment with the CCSG COE criteria. For the CCSG criteria focused on CA definition, CA characterization, engagement of CA communities on cancer research and cancer control efforts, and engagement of Center members on community needs, the short-term objectives are to increase: (1) center staff and researcher awareness/knowledge of the CA priorities and needs, (2) research and extramurally-supported awards on CA populations, (3) educational programs offered at CAs, (4) representation of CA populations on research studies, and (5) community awareness of cancer needs. For the CCSG criteria focused on the engagement of center members on community needs, and the center’s cancer control efforts, the short-term objectives are to increase: (1) the number of minoritized and marginalized communities reached/educated relative to cancer prevention and risk reduction, (2) cues to action, including people and things that activate behavioral change, (3) knowledge of participants relative to cancer prevention and control, (4) cancer awareness, (5) positive attitude relative to cancer prevention and risk reduction, and (6) behavioral intention relative to cancer prevention and risk reduction.

For the CCSG criteria focused on facilitating accrual to clinical trials, the short-term objectives are to increase: (1) the number of minoritized and marginalized communities reached/educated relative to clinical trials, (2) cues to action that activate accrual to clinical trials, (3) clinical trials knowledge of participants, (4) clinical trials awareness, (5) positive attitude about participating in research/clinical trials, (6) behavioral intention relative to participating in research, (7) positive attitude about donating samples for research, and (8) behavioral intention relative to donating samples for research. For the CCSG criteria focused on expanding the center’s reach, the short-term objectives are to increase: (1) research collaborations with investigators from Low- and Middle-Income Countries (LMICs), (2) global oncology research and extramurally supported awards; (3) global oncology research training in LMICs, and (4) global oncology research capacity in LMICs. The COE-IM includes a strong program evaluation to evaluate the impact of MCCCC COE objectives for continuous quality improvement.

### The MCCCC COE Logic Model: foundation for continuous improvement

#### Role of program evaluation in facilitating the operations of COE

In order to gain an in-depth understanding of the impact of COE initiatives, program evaluation frameworks in public health are often used to document and monitor the progress of activities. From a utilization-focused evaluation approach, program evaluation is valuable to facilitate the operations of COE by 1) identifying a program’s goals and priorities 2) improving overall program effectiveness, and 3) guiding future decision-making [4]*.*

##### Identify priorities

A formative evaluation can be useful in the initial states of program development, in determining specific needs, establishing processes and plan data collection in preparation for implementation [5]. For example, as a MCCCC COE initiative, the cancer-focused Community Needs Assessment (CNA) was developed by an interdisciplinary team of scientists to understand CA needs and priorities. As results of the cancer-focused CNA are reported, a feedback approach for responding to community’s needs will be developed through program evaluation. The cancer-focused CNA incorporates population and sociocultural constructs to account for diverse groups in CAs and will be instrumental in shaping programs to reduce cancer burden in marginalized groups.

##### Improve effectiveness

Summative evaluation helps to determine impact of a program, if the goals and objectives of the program are being met, and if the program accomplished what was originally intended [6]. To improve effectiveness, outcomes are assessed, and strategic decisions are made to modify program components to better meet the needs of the community. As an approach to enhance current efforts and maximize impact in COE, a training curriculum on COE evidence-based intervention was initiated, based on program planning frameworks. First, an inventory of current COE programs was assessed and categorized with preliminary evaluation outcomes. Second, programs with highest impact and strongest partnerships were prioritized for re-implementation. Third, management staff underwent training for evidence-based planning, implementation, and evaluation, and developed operational plans for evidence-based program implementation in their subject areas of expertise. Focused training on evidence-based interventions and practices in promoting cancer health equity has strong potential to improve effectiveness of programs by integrating scientific rigor in COE efforts, developing evaluative measures to document progress and demonstrate accountability, and building capacity for established and active community partners invested in reducing cancer health disparities.

##### Guide decision-making

Following implementation, the results of the evaluation can include a) summary of overall outcomes relative to program goals and objectives, b) highlights of accomplishments, c) progress towards goals of specific programs, and d) recommendations for improvement that are communicated to both internal and external audiences and stakeholders for ongoing program improvement. This aspect of program evaluation is particularly useful to facilitate evidence-based decision-making. To foster collaborative relationships, community feedback is interwoven in planning for program improvement and improves agency of local partners in COE efforts. In COE operations, program evaluation is vital in gaining understanding of strategies for sustainable, scalable programs to reduce unnecessary costs and efforts, improving community health disparities, monitoring overall progress, and maximizing impact of collaborative evidence-based programs. With an effective evaluation plan, outcomes based on predefined goals can be carefully monitored and tracked, yielding valuable insight on achievements and gaps on progress.

#### Use of Logic Model in operationalizing Impact Model

**Fig. 2:**
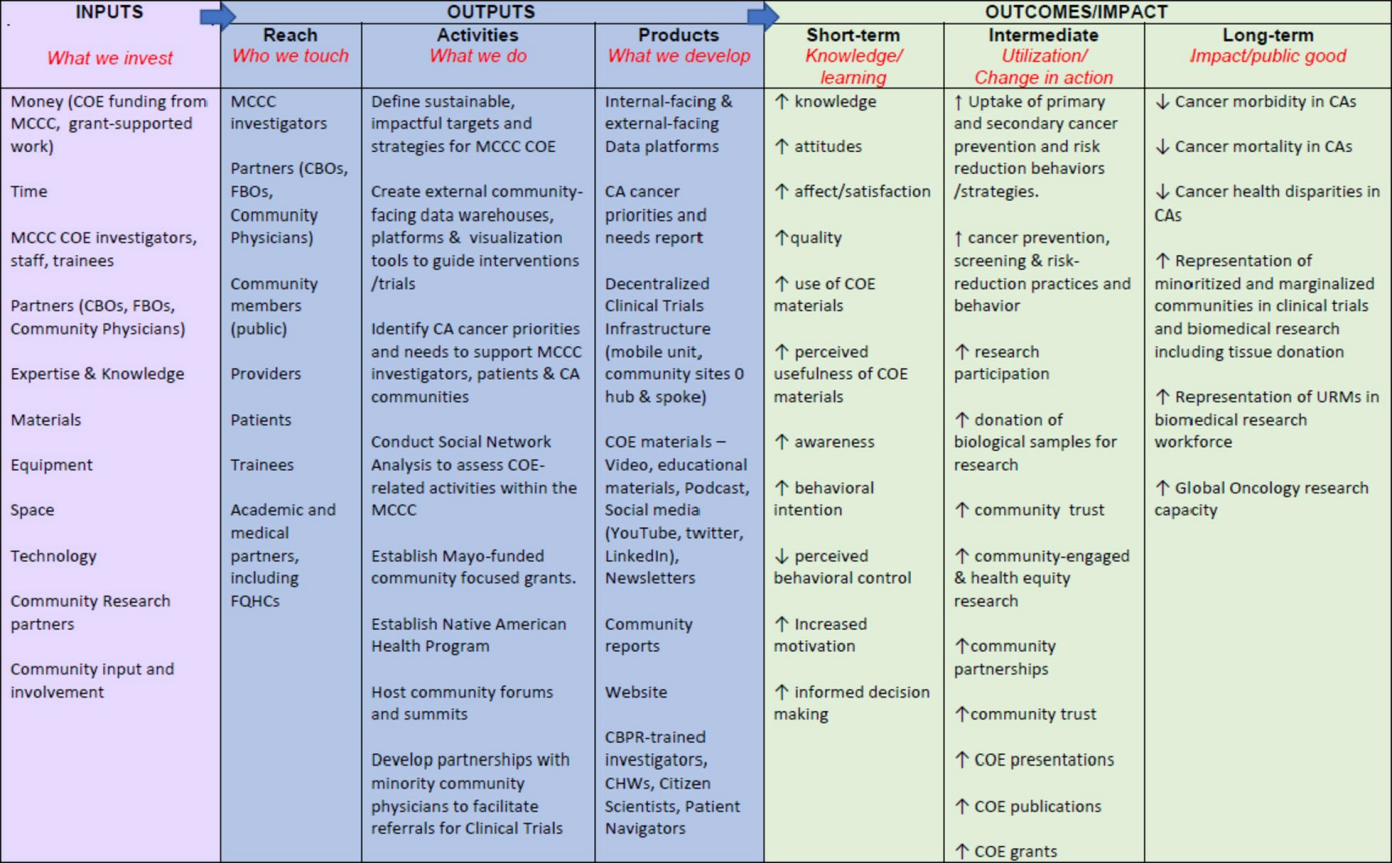
Mayo Clinic Comprehensive Cancer Center Community Outreach & Engagement (MCCCC COE) Logic Model developed to identify objectives, and track and monitor activities for process improvement of COE activities

Logic models are used in tandem with evaluation plans, often as a blueprint for programs’ resources, activities, and expected outcomes. A logic model can outline *what* a program is trying to accomplish, *how* to pinpoint specific areas of assessment that is most needed, and at *which* timepoints evaluation tools should be disseminated [7]. Driven by theories of change, a logic model is used to identify clear objectives and measure expected results of a program or initiative to improve impact and value [8]. To operationalize the aims defined in the COE-IM, a Logic Model was developed in line with the MCCCC COE goals for CA communities. The logic model was developed in line with the CCSG guidelines, driven by MCCCC long-term goals, which reflect significant efforts to decrease cancer disparities in risk factors, incidence, prevalence, morbidity and mortality, and increase access to appropriate cancer education, resources, and care particularly for minoritized and marginalized populations. The COE Logic Model (see Fig. 2) visually depicts the sequence of progress towards long-term impact and overall goals for MCCCC COE initiatives.

The *inputs* include items invested (time, money, staff equipment, etc.) and needed to generate *outputs* focusing on the *reach* of specific populations, partners, and groups, through various *activities*, to develop tangible *products* which are instrumental in operations towards achieving goals. The *short-term goals* describe the intended changes in knowledge and learning, while the *intermediate outcomes* are expected over a longer period and focus on the utilization or changes in action. The *long-term outcomes* are aligned overall goals of MCCCC COE and outline the societal impact of COE programs in the CA communities.

#### MCCCC COE inputs

Identifying the resources available and investments required to operationalize the COE-IM represents the foundation of the COE Logic Model. The inputs, what MCCCC COE invests in both quantity and quality, determine the success and sustainability of the programs. In addition, this foundational process reveals gaps in resources and the barriers or constraints that require identifying critical strategic factors to overcome [9]. For the COE Logic Model, the inputs are: (1) money; (2) time; (3) personnel; (4) partners; (5) expertise & knowledge; (6) materials; (7) equipment; (8) space; (9) technology; (10) community research partners; and (11) community input and involvement. The following descriptions of each input are provided.

##### Money

MCCCC COE programs, as a part of an NCI-designated comprehensive cancer center, are funded from the NCI CCSG award, non-CCSG grant awards, institutional and philanthropic funds. In addition, COE has been identified as a strategic priority within MCCCC for potential benefactors by its development team.

##### Time

The COE Logic Model reflects three different considerations for time: time needed to build community trust; sequencing of inputs and activities to produce desired outputs; and timelines/milestones that measure progress towards identified strategic aims. The COE transition team established two-year and five-year objectives, which served as the initial filter for inputs required in the short term versus long term.

##### Personnel

A key strength of the MCCCC COE personnel is the diversity of background, thoughts and worldview that represents our CA populations. The COE Office and Programs is directed by an enterprise-wide Deputy Director, with Associate Directors designated for each of the three sites in Arizona, Florida and the Midwest. There is also COE Faculty members, comprising 9 faculty with expertise in community engagement research. The COE Operations team is led by an Operations Administrator and a COE Director at the enterprise-level. Each site has a project manager, outreach coordinators and trainees.

##### Community input and involvement

According to the NCI CCSG guidelines, Community Advisory Boards (CABs) are crucial to ensure bi-directional communication as well as receive consistent and timely feedback from the community [10]. MCCCC COE has a CAB comprised of a diverse group of community leaders, cancer survivors, and advocates representative of its CA. The MCCCC COE CAB serves as a connector between the MCCCC and the CA communities served by the MCCCC through community engagement, partnerships, and outreach. Representing the rich diversity of the MCCCC CA populations, CAB members are thought leaders that advise, prioritize, and participate in the development of solutions that include community-engaged research, cancer prevention and control activities, and clinical care efforts to address the cancer needs of our diverse catchment area population. In addition to the CAB, community members have the opportunity to be involved with researchers through the MCCC COE’s Community Engagement (CE) Studios [11] and Citizen Scientist program.

##### Partners

Partnership is a key input of the MCCCC COE. The COE Office and Programs strategically forms partnerships that are long term and bi-directional, including formal and informal coalitions and collaborations. The MCCCC COE has over 200 partners that range from community-based organizations, faith-based organizations to community health systems that serve minoritized and marginalized communities, including federally qualified health centers.

##### Expertise

Apart from academic and professional knowledge in relevant areas, passion for social justice is a common thread across both external and internal stakeholders. Additional skills and abilities held by stakeholders include emotional intelligence, organizational, management, interpersonal and communications, which are all skills vital to engaging and meeting the needs of the MCCCC CA communities.

##### Equipment and materials

The MCCCC COE Office and Programs provides shared resources for the community and MCCCC members nationally and globally. As an example, MCCCC has provided digital signages, computers and large screen monitors at the community level to develop COE resource center hubs in several community sites. Equipment that has been provided include freezers and ultrasound equipment for biobanking at international sites, as well as solar panels for electricity in low resource settings.

##### Space

The nature of community outreach and engagement work requires reaching the CA populations where they are. It is thus imperative that MCCCC COE has a consistent presence where individuals live, work, and reside. In Florida, Mayo Clinic established the Mayo Clinic Community Health Collaborative (MCCHC) within the Jacksonville community as a community-based resource center to foster outreach, education and community engagement research, including minimal-risk clinical trials. Across the enterprise, we have partnered with multiple organizations with physical presence in minoritized and marginalized communities using a hub and spoke model. This includes the American Legion Post, Urban League, YMCA and County extension offices.

##### Technology

The use of technology to reach minoritized and marginalized communities is a critical tool to address cancer health disparities. However, this must be done in such a way that it does not create a digital divide for the CA communities. MCCCC COE input in this area includes setting up digital health platforms and video communication platforms at community COE spokes. In addition, MCCCC COE is currently working with the Mayo Clinic Platform team to develop and make public the profiles of CA counties, including population demographics, socio-demographic factors, behavioral risk factors, cancer screening, and cancer burden and cancer patterns. Preliminary profiles have been successfully used to support COE mini grant applications by community-based organizations. All applicants were provided the profiles for the CA counties targeted for their applications, making it easier for the applicants to target the needs of their communities.

#### MCCCC COE reach

Community stakeholders impacted by MCCCC COE ranges vastly across both the internal and external environment. Examples of the COE reach, or who the Center touches are described below.

##### MCCCC programs and members

**Fig. 3:**
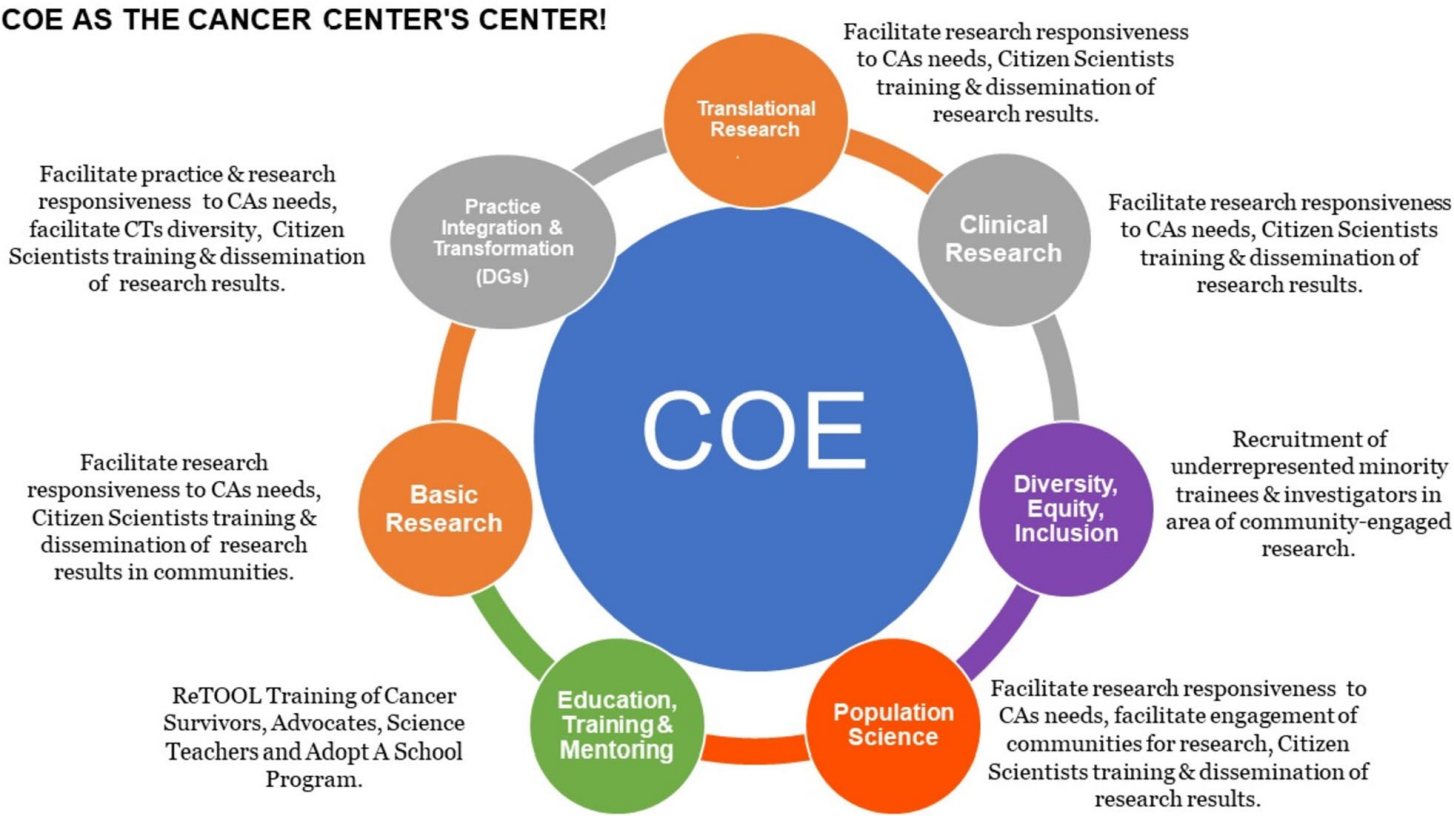
Various activities conducted by Community Outreach & Engagement (COE) which provides support for Mayo Clinic Comprehensive Cancer Center (MCCCC) members for community-engaged, community based participatory, health equity, health disparities, and/or population health research

MCCCC COE supports MCCCC members for community engaged, community-based participatory, health equity, health disparities, and/or population health research through multiple activities, including monthly help sessions and tailored consultative services. Additionally, the COE Office and Programs works closely and synergistically with other MCCCC Offices and Programs. Figure 3 provides a summary of these partnership activities.

##### Community partners

MCCCC impacts individuals, families, groups, community organizations, and private institutions within its CA, spanning 75 counties across five states. MCCCC COE aligns its interventions and activities across the cancer continuum, from prevention to survivorship and advocacy, to maximize its impact and engage these partners in the research process. MCCCC COE partners include clinical partners, faith-based organizations, and community-based organizations.

##### Community members (public)

MCCCC is well represented by its CA communities through the COE administrators, faculty, staff and CAB members. In addition, the COE conducts a cancer focused needs assessment to identify the CA’s needs and priorities that augments the federally required Community Health Needs Assessment (CHNA). These community inputs allow MCCCC COE to address identified issues, that touches the public, to improve community health and reduce the cancer burden. For example, in Florida, MCCCC COE partnered with the American Legion Post #197 and the Jacksonville Urban League to establish community infrastructure supportive of community research and initiatives.

##### Providers

The strength of Mayo Clinic lies in its ability to address serious and complex health conditions. One of MCCCC COE’s priorities is thus reaching the health professionals and organizations that serve CA communities with opportunities to participate in critical research that contributes to possible health cure. As an example, MCCCC COE partnered with The Way Clinic, Barnabas, Aza Health, and Flagler Free Clinic to increase outreach and research activities in rural Florida.

##### Patients/survivors

The COE activities cover the cancer care continuum, including outreach for survivorship care that are culturally and ethnically appropriate, and inclusive research for patients and survivors.

##### Trainees

MCCCC COE supports underrepresented students, interns, postdoctoral fellows, and early-stage investigators with opportunities to learn about and be involved in community-based research. In collaboration with MCCCC Cancer Research Training and Education Coordination (CRTEC) and Diversity, Equity and Inclusion (DEI), the Office also organizes webinars and workshops focused on community engagement research and community outreach initiatives.

##### Academic and medical partners

Collaboration with other academic and medical institutions enables MCCCC COE to significantly impact the cancer burden within its CA. Universities, research institutions, and Federally Qualified Health Centers as partners in research studies and clinical trials acts as a force multiplier to address public health concerns that affect everyone. Examples of MCCCC COE academic and medical partners are Adelante Healthcare (AZ), Community Health Services, Inc. (MN), and Agape Health (FL).

#### MCCCC COE activities

The MCCCC COE activities to reach populations, partners, and groups in the CAs, include activites developed through an iterative process that includes internal and external stakeholders. Examples of these activities are provided below.

##### Creation of external community-facing data warehouses, platforms & visualization tools to guide interventions /trials

The community facing platform provides community members and external stakeholders with information about the cancer burden that impacts the 75 counties across the MCCCC CAs. The community cancer profile by county includes county demographics, cancer incidence and mortality and cancer screening rates. MCCCC COE is working with CA communities to develop and implement activities that address the cancer burden in the CA counties.

##### Identifification of CA cancer priorities and needs to support MCCCC investigators, patients & CA communities

The Cancer Needs Assessment is currently being dissemenated throughout the MCCCC CAs. There are several measures, which includes, demographics, cancer family history, lifestyle behaviors, and social determinants of health. The assessment utilizes several methods of data collection, including push to web surveys, survey panels, as well as, in-person data collection.

##### Established MCCCC-funded community mini-grants

The primary goal of the MCCCC COE Community Mini-Grant program is to facilitate the elimination of cancer health disparities by reducing cancer burden in the communities served by Mayo Clinic. The Mini-Grant awards up to $20,000 to community organizations for cancer focused projects. Successful awardees work closely with the MCCCC COE Office to implement evidence-based intervention programs to improve health at the individual, community and/or health system level.

##### Established Native American Health Program

The Native American Health Program includes culturally tailored activities aimed at improving the cancer burden in the Native American communities served by MCCCC COE. Specific activities include tribal focused cancer needs assessment and partnership building with community organizations that serve Native Americans.

##### Community forums and summits

MCCCC COE regularly hosts culturally tailored community forums and summits focused on cancer-related topics across the cancer care continuum. This includes the Cancer Conversations series that features medical experts and survivors providing information about different cancers. The annual Mayo Clinic Partnership Summit brings together MCCCC members and community members to foster relationship building, engagement, and bi-directional communication for engaging community stakeholders.

##### Partnerships with community providers to facilitate referrals for clinical trials

MCCCC COE continues to build partnerships with community clinical providers, including those in private practice, community health centers, as well as Federally Qualified Health Centers. The goal of the partnerships is to understand the specific cancer needs of their patients, the research interests of the community providers, and the capacity of providers to partner on the development of a decentralized clinical trials model that will facilitate increased community access to MCCCC clinical trials. One example of this is MCCCC COE’s partnership with the Northeast Florida Clinical Trials Consortium, which is made up of Black physicians serving patients in Northeast Florida.

#### Description of MCCCC COE products

Examples of the COE Office and Programs products are summarized below.

##### Internal-facing & external-facing data platforms

The interactive platforms provide internal and external stakeholders with a variety of data, including CA demographics and cancer burden by county (incidence/mortality rates, cancer risk behaviors, screening rates, etc.), internal and external resources to address social determinants of health.

##### Catchment area cancer priorities and needs report

The MCCCC COE Community Cancer Focused Needs Assesment survey is currently ongoing with data collection in CA counties. The results of the survey will be analyzed to determine the community level needs across the MCCCC CA. The analysis will be developed into an internal report that will be disseminated to MCCCC members to faciliate clinical and research activities, as well as, a community report that will be disseminated externally to facilitate the development of activities to address the identified needs and priorities.

##### Decentralized Clinical Trials infrastructure

In an effort to faciliate increased community access to clinical trials, the Decentralized Clinical Trials infrastructure is a “hub and spoke” model. In this model, MCCCC sites serve as the hub of clinical trial implementation (i.e. development of trials, regulatory activites, primary study coordination, etc.). The spokes of the model include resources within the CA to improve access. Examples of the spokes resources are:

- *Mobile Research Unit (MRU):* The MRU is an RV-style vehicle that is a self-contained, mobile research facility. It includes two exam rooms, equipment for lab tests, a private area for participant interviews, audio/visual technology for participant education, and a laboratory. The Northeast Florida based unit is used to bring minimal risk research studies to rural and hard-to-reach communities thereby expanding access to research studies.
- *Community Based Sites:* The community sites serve as locations for varying clinical trials, including, but not limited to, recruitment, research interventions, and trial follow-up activities. The sites range from community practices, Federally Qualified Health Centers, Community Health Centers, and other community based organization locations.

##### COE multimedia materials

MCCCC COE disseminates information to internal and external stakeholders addressing cancer across the continuum, foster bi-directional communication between community stakeholders and MCCCC members to faciliate research participation, and provide updates on COE activities and results. Materials are disseminated through community reports, websites, social media, print materials (newsletters, infographics, etc.), and video format.

##### Community-based training programs

In an effort to faciliate research participation in MCCCC research activites, COE has developed several training programs for MCCCC members and communities. For example, COE developed a COE Faculty training program focused on Community-Based Participatory Research (CBPR), cultural humility and responsiveness to successfully engage, recruit and retain diverse research participants. COE also developed a Citizen Scientist Program and Community Health Worker (CHW) Cancer Training Program to educate and empower community members to engage in the research process and understand key cancer topics across the cancer contiuum.

#### MCCCC COE short-term and intermediate outcomes

When examining the short-term outcomes and impact of COE efforts, the measurements involve the extent of change primarily in knowledge and learning. Expanding the reach of COE can ultimately impact awareness, utilization, and representation of cancer-focused resources, particularly in the CAs. The impact of outcomes in the short-term include increase in individual-level factors such as *knowledge, attitudes,* and *satisfaction* regarding general understanding of cancer disparities and available resources. Additionally, the quality, use, and perceived usefulness of COE materials are expected to increase. Finally, short-term impact can also include an increase in awareness, behavioral intent, perceived behavioral control, motivation, and informed decision-making relative to cancer health and clinical care. To measure the change in knowledge and learning, surveys, interviews, focus groups, data analytics and self-reports of education and attitudes over a period are documented and monitored.

As knowledge and learning is strengthened, it leads to changes in action or utilization, such as increasing primary and secondary cancer prevention and risk reduction behaviors, screening, and practices. There is also increased likelihood for research participation, and donation of biological samples for research, so that clinical needs can better serve minoritized and marginalized communities. Employing more community-focused approaches also increases the potential for community trust, partnerships, and community engaged research. Improved collaboration with invested partners will increase overall research scholarship in the field, with increased number of 1) underrepresented minorities (URMs) in biomedical research; 2) COE presentations, publications, and grants; 3) focused community reports; and 4) community-engaged and health equity research led by MCCCC investigators. These intermediate outcomes are tracked through documentation of participation, self-reported behaviors, and feedback from community partners. Teams led by the operations team are implementing evidence-based interventions with guided evaluation plans in context-specific CA settings to determine reach, impact, and value of programs and best practices for future directions.

#### MCCCC COE long-term outcomes

The final component of the logic model describes the long-term outcomes and impact of the MCCCC COE, which includes decrease in cancer morbidity, mortality, and disparities in the CAs measured over time by the cancer incidence, prevalence and mortality rates in CA communities. It is also expected that there will be an increase in the representation of minoritized and marginalized communities in clinical trials, biomedical research, and tissue donation to effectively address cancer health disparities in CAs. Additionally, increasing the representation of URMs in the biomedical research workforce expands diversity in research and clinical practice. Finally, the MCCCC COE is increasing global oncology research capacity in LMICs to address the burden of cancer globally.

## Discussion

The implementation of the COE-IM and Logic Model has led to increase in COE innovations at the MCCCC, facilitating our progress to effectively address the 2018 CCSG critiques. Examples of some of the innovations are provided below.

### Responsiveness to CAs cancer priorities and needs

One of the key initiatives of the MCCCC COE is the Community Mini-Grant (CMG) program. Established in 2022, the CMG program was launched to strengthen partnerships between MCCCC and community-based organizations in CA communities. The primary goal of the COE CMG program is to facilitate the elimination of cancer health disparities by reducing cancer burden in the communities served by the MCCCC. For the 2022/2023 award year, the program was limited to projects that specifically address cancer focused community health needs. All potential applicants were provided access to the cancer profiles of the CA communities targeted for the CMG application. Thirteen (13) applications were received for the 2022/2023 award year with a total of six funded across the CAs. Table 2 summarizes the successful organizations, project focus and location. Successful awardees are working closely with the MCCCC COE to implement proposed programs with the goal of improving health at the individual, community and/or health system level.

**Table 2:** Community Mini-Grant (CMG) program awardees

**Organization**	**County**	**Title**	**Amount Requested**
Faith Missionary Baptist Church	Maricopa, AZ	Wellness Vision Plan	$20,000
New Home Baptist Church	Maricopa, AZ	Novel Strategies to Increase Awareness about Melanoma	$20,000
The Way Clinic	Clay, FL	Center of Excellence for Cancer Literacy and Clinical Trials	$20,000
125 Live	Olmsted, MN	Cancer Survivor Fitness Program	$14,748
Hispanic Advocacy and Community Empowerment through Research	Mankato, MN	Bringing Educative Breast Cancer Detection Programs	$20,000
Village Agricultural Cooperative	Olmsted, MN	Cancer Prevention through Reduced Sun Exposure and Community Education	$20,000

### Scientific discoveries relevant to CAs priorities and needs

To catalyze research that is responsive to the needs of the CA communities in all areas of science, the COE developed a CA communication campaign and consulting services with the goal of increasing MCCCC members’ awareness of the CA priorities and needs. Multiple programs have been implemented/proposed to support MCCCC members in developing research programs responsive to the needs of the CA communities, including: (1) the MCCCC Office of Communications communique on CAs sent to MCCCC leadership, non-MCCCC Mayo Clinic leadership, and MCCCC members, (2) assignment of liaisons from each MCCCC Research Program and Disease Group to COE, (3) assignment of COE faculty to each MCCCC Research Program and Disease Group, (4) provision of tailored COE consultation services to MCCCC members, (5) monthly COE help sessions for MCCCC members, and (6) COE enterprise-wide and site specific Town Halls to educate MCCCC members on CA priorities and needs, as well as share information about COE infrastructure and resources for MCCCC members.

### Patient and minority access and accrual to trials and interventions

Multiple initiatives have been implemented to foster accrual to clinical trials from CA communities. An initiative that has been very successful is the Community Clinical Trials Champion program. Launched in 2022, the program includes clinical trials education and enrollment of interested community participants in the Mayo Clinic Community Research Registry (CoRR). The CoRR is a community-based registry of minoritized and marginalized populations who consent to be contacted for biomedical research including clinical trials and bio-specimen donation. The database serves as sampling frames for the recruitment of underrepresented adults in biomedical research/clinical trials, who initially meet specific study eligibility criteria. CoRR participants provide written informed consent (authorization) to allow Mayo Clinic to include their contact information along with certain self-reported health information related to chronic diseases to be placed in the registry. Mayo Clinic partners with faith-based organizations, sororities, fraternities and other community-based organizations to oversight, recruit and administer the CoRR program. The data is collected using REDCap, and includes name and contact information of participants, demographic information, chronic disease status and explicit consent to be contacted for future research studies (with an option to state the type of study).

### Hub and spoke model to address CAs cancer needs

The MCCCC CAs span several counties in Arizona, Florida, Midwest and across multiple health systems nationally. Developing effective cancer control efforts to reduce the burden of cancer in the MCCCC CA communities thus requires meeting our populations where they are. In partnership with the CAB, a hub and spoke model was developed to provide evidence-based cancer prevention and control services in the MCCCC CAs. For example, in Florida, a Mayo Clinic Community Heath Collaborative (MCCHC) has been established in the Health Zone 1 region of Duval County. Health Zone 1 has the largest number of minority populations in Duval County and experience significant health disparities. Serving as a health hub, the MCCHC is affiliated with multiple collaborative health spokes in Northeast Florida, including the American Legion Posts, extension offices and the Urban League to facilitate collaborative initiatives on cancer prevention, risk reduction, and recruitment for cancer clinical trials. The American Legion Post 197, also located in Health Zone 1, has emerged as an important health spoke in reaching CA populations in minoritized communities. With a mission to enhance the well-being of America’s veterans, their families, military, and communities through devotion to mutual helpfulness, the American Legion is the nation’s largest wartime veterans service organization aimed at advocating patriotism across the United States (US) through diverse programs. The American Legion has more than 12,000 Posts in communities throughout the US that provide potential opportunities to reach diverse populations in their communities. The American Legion Post 197 primarily has minority veteran members and opened its community health resource center to the public in 2022 with support from Mayo Clinic and the MCCCC. Although the Mayo Clinic-American Legion Post 197 collaboration started in 2021, it includes multiple cancer research projects funded by the US Department of Defense.

## Conclusion

The recent finding by Alaniz and Rebbeck [12] indicating a strong correlation between COE component merit descriptors and the overall CCSG cancer center review score underscores the importance of this paper. The two guide models developed by the MCCCC COE Office and Programs, the COE-IM and Logic Model, has facilitated the COE’s responsiveness to the CCSG requirements to meet the needs of the MCCCC CA communities. These models can be adapted by other cancer centers or institutions to meet the needs of their communities in achieving health equity for all.

## Data Availability

The authors confirm that all data generated or analyzed during this study are included in this published article.

## Abbreviations

AZ: Arizona
CA: Catchment Area (CA)
CAB: Community Advisory Board
CNA: Community Needs Assessment
CBPR: Community-Based Participatory Research
CCSG: Cancer Center Support Grants
CHNA: Community Health Needs Assessment
CHW: Community Health Worker
COE: Community Outreach and Engagement
COE-IM: COE Impact Model
CMG: Community Mini-Grant
CoRR: Community Research Registry
CRTEC: Cancer Research Training and Education Coordination
DEI: Diversity, Equity and Inclusion
FL: Florida
LMICs: Low- and Middle-Income Countries
MCCCC: Mayo Clinic Comprehensive Cancer Center
MN: Minnesota
MRU: Mobile Research Unit
NCI: National Cancer Institute
U.S.: United States
